# Codon usage bias analysis of genes linked with esophagus cancer

**DOI:** 10.6026/97320630017731

**Published:** 2021-08-31

**Authors:** Hemashree Bordoloi, SR Nirmala

**Affiliations:** 1 Deptartment of Electronics and Communication Engineering, Gauhati University, Assam, Indi; 2 Department of Electronics and Communication Engineering, Assam Don Bosco University, Assam, India; 3 School of Electronics and Communication Engineering, KLE Technological University, Karnataka, India

**Keywords:** Natural selection, mutation pressure, compositional constraints, RSCU

## Abstract

Esophageal cancer involves multiple genetic alternations. A systematic codon usage bias analysis was completed to investigate the bias among the esophageal cancer responsive genes. GC-rich genes were low (average effective number of codon value was 49.28). CAG and GTA are over-represented and under-represented codons, respectively. Correspondence analysis, neutrality plot, and parity rule 2 plot analysis confirmed the dominance over mutation pressure in modulating the codon usage pattern of genes linked with esophageal cancer.

## Background:

Due to the degeneracy of the genetic code, we observe significant variation in synonymous codon usage in the coding sequences [1]. Mutation is the prime source of synonymous codon usage variation where selection pressure decides their selection and adaptation [2,3]. Supek et al 2014, revealed the role of synonymous mutation in cancer progression [4]. Later on, it became evident that CUB and synonymous mutations have a non-trivial effect on human diseases [5]. Reports on the synonymous variant of genes having a role in metabolic processes revealed that CUB information may increase the diagnostic accuracy in a variety of diseases [6-8]. Cancer is a genetic disorder that results from catastrophic mutations causing genetic alternations [9]. Among the different cancer types, esophageal cancer (EA) ranks as seventh and sixth serious malignancy with respect to prognosis and mortality rate, respectively [10]. The 5-year survival rate of EC patients is within the range of 15% to 25% [10]. The incidence rate is higher in developing countries from the Asian region (highest in China) [11]. According to the Indian Council for Medical Research (ICMR), the number of EC patients is increasing each passing year in India [12]. Several independent research done on the molecular changes linked to EC have identified key regulatory genes of EC [11,13]. Therefore, it is of interest to document the codon usage bias analysis of genes linked with esophagus cancer.

## Materials & Methods:

### Sequence data:

We identified 82 human genes with a role in EC. The complete coding sequence of these genes was retrieved from the NCBI nucleotide database (www.ncbi.nlm.nih.gov). The sequences were pre-processed with an in-house Perl script to check sequences taken for CUB analysis starts with a proper initiation, ends with termination codons, and are perfect multiple of 3 bases.

### Effective number of codons (ENc):

Wright proposed ENc in 1990. It quantifies the degree of CUB in a given sequence [14]. The values of ENc values can vary from 20(extreme bias where only one codon is used per amino acid) to 61 (without bias where codons are used in equal probability). ENc was calculated as follows: (see PDF)

### Relative synonymous codon usage (RSCU):

RSCU is the observed frequency of a codon divided by the expected frequency [15]. If all synonymous codons encoding the same amino acid are used equally, RSCU values are close to 1.0, indicating a lack of bias. Moreover, the codon with RSCU value greater than 1.6 is treated as over-represented codon, whereas the codon with RSCU value lower than 0.6 is considered as underrepresented codon. RSCU value of a codon is estimated as: (see PDF) 

### GC content analysis:

To quantify the variation in base frequencies that occur in varying numbers at each codon site we calculated GC1s, GC2s & GC3s i.e. frequency of GC content at first, second, and third codon positions, respectively. (see PDF)

### Neutrality plot:

Reports suggest that there is a bias in the rate of mutations at three different codon positions, particularly high at the synonymous third codon position. Theoretically, mutation should occur randomly if there is no external pressure. The preference of bases in three different codon positions is not the same in the presence of selection pressure [16]. Neutrality plot, a graphical plot of GC12 against GC3 depicts the roles of directional mutational pressure against natural selection.

### Parity rule 2 (PR2) plot:

PR2 bias plot was generated by plotting the [G3 /(G3 + C3)] vs [A3 /(A3 + T3)] [17]. In the PR2 plot, the center region coordinate is (0.5, 0.5). If a gene shows its presence on the center then it suggests that A = T and G = C and no bias is the composition of the gene.

### Correspondence analysis:

Correspondence analysis has been successfully used to explore codon usage variation among genes [18]. It is a commonly used multivariate statistical technique, in which all genes were plotted in a 59-dimensional space, according to the usage of the 59 sense codons (excluding codons for Met, Trp, and stop codons). The plot was then used to identify the axes, which represent the most prominent factors contributing to variation among genes.

###  Software used:

Codon-pair context quantifications were performed using Anaconda 2 [19]. The association of codon pairs was assessed using the chi-square test of independence. All the statistical analyses were done using the SPSS software (version 16.0). Acua software was used to find the nucleotide composition at synonymous positions, compositional skewness, CAI, and ENc values [20]. RSCU value was calculated using INCA software [21]. Correspondence analysis was done using past3 [22]. CodonW was used to calculate GRAVY and Aromo values (http://codonw.sourceforge.net/).

### Codon usage bias:

ENc value was used to determine the overall codon usage bias. From Table 3(see PDF) we observed that ENc value of the EC-related genes ranges from 33.778 to 56.976, with a mean ± standard deviation of 49.28 ± 5.72. This low bias (ENc<35) in codon selection might have a link with DNA replication as these genes are actively synthesized in different cell types. Moreover, we observed a significant negative correlation between ENc and GC3 (r=-0.56, p<0.05). This further suggests the impact of GC nucleotide composition on the CUB. Genes with high GC3 composition showed the highest CUB. However, the correlation coefficient did not reach the extreme value i.e. 1, which further suggests that GC3 is not the sole determinant of CUB confirms the variation of nucleotides and thus CUB in EC-responsive genes. RSCU analysis further showed the dominance in the use of a specific group of codons in EC-responsible genes. 28 codons out of 60 codons showed RSCU score greater than 1, 7 codons RSCU > 1.6, whereas, 9 codons RSCU < 0.6 (Table 4 - see PDF).

## Results:

### Nucleotide Composition:

Nucleotide composition analysis was performed to identify the compositional variation present in the genes responsible for EC. From our analysis, it was observed that the EC-responsive genes are GC-rich (52.01%), similar to the overall genomic composition. Positional bias analysis revealed that in 59% gene G3>A3, whereas in 7% gene A3 is equal to G3 and in 34% gene A3>G3. Similarly, in 72% gene GC3>AT3, only in 1% gene AT3> GC3, whereas, in 27% gene AT3>GC3. This indicates the dominance of GC composition at synonymous codon positions. AT3 was found to be highest in the RBBP6 gene and GC3 in the SOX17 gene. Table 1 represents the nucleotide composition of different genes responsible for EC. Furthermore, we observed a strong significant correlation between homogenous and heterogeneous nucleotide compositions (Table 2 - see PDF). These intricate correlation patterns suggest the influence of mutation (major) and selection pressure (minor) is shaping the CUB of EC-responsive genes. Our findings corroborated with the results reported elsewhere [23].

### Variation in codon usage:

**Figure 1:**
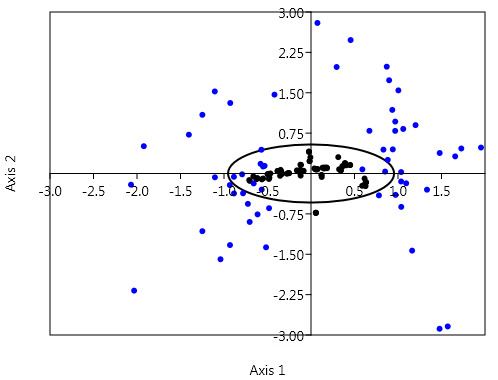
Correspondence analysis (COA) of EC- responsible genes based on the RSCU score of 59 codons. Black color indicates genes, blue-colored dots represent synonymous codons. Axis 1 and axis 2 contributes 27.5% and 11.6% of the total variation, respectively

**Figure 2:**
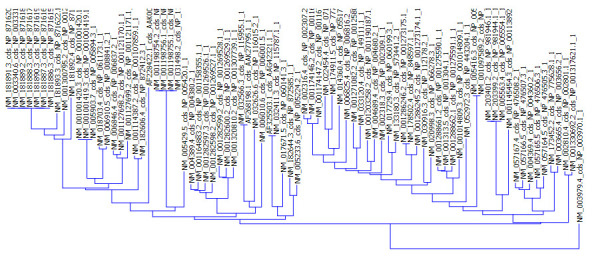
Cluster analysis for genes responsible for esophageal cancer. The gene cluster was constructed based on the neighbor joining method.

To explore the variation in synonymous codon usage we did correspondence analysis based on the RSCU score of 59 codons across the 82 EC-responsible genes. Axis 1 and axis 2 were found to be the major contributors i.e. 27.5% and 11.6% of the total CUB variation. In Figure 1 black dot represents the genes and the blue dots represent codons. From Figure 1 it is evident that the majority of the codons are close to the two axes, whereas, genes were found to be near the center region. These findings altogether confirm the relative contribution of nucleotide composition under the influence of mutation pressure (major) in shaping the observed codon usage pattern [24]. Next, we did cluster analysis to validate the findings of our correspondence analysis (Figure 2). The findings of our cluster analysis corroborated well with the COA result.

### Neutrality plot analysis:

**Figure 3:**
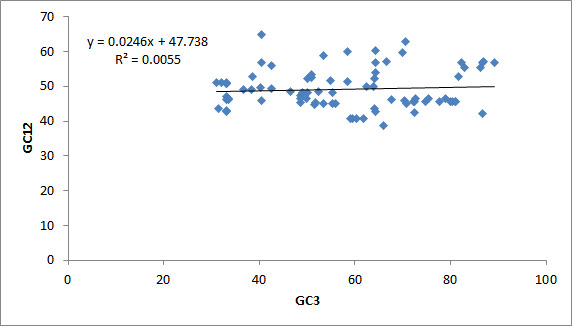
Neutrality plot analysis for genes responsible for esophageal cancer.

To evaluate the contribution of two major forces in shaping the codon usage pattern of EC-responsible genes we plotted GC12 vs GC3. We observed a significant positive correlation between them (r=0.684, p<0.01). The regression coefficient of GC12 on GC3 is 0.0246 (Figure 3). Previous reports suggest that if the regression coefficient in the neutrality plot is greater than 0.5 then there is a significant contribution of mutation pressure [25]. However, here we observed the opposite thereby our neutrality analysis suggests the dominance of selection pressure in shaping the CUB of EC-responsible genes.

### Parity rule 2 (PR2) bias plot analysis

**Figure 4:**
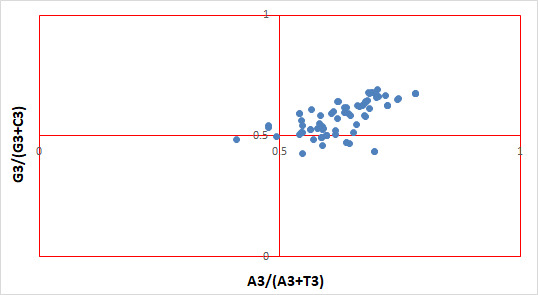
PR2 plot of genes responsible for esophageal cancer.

Mutation forces the random use of nucleotides at the synonymous codon position whereas selection pressure does not force the equal use of nucleotides [26]. In the PR2 plot, if the genes coincide in the center region then it suggests the dominance of mutation pressure whereas deviation from the center indicates the contribution of selection pressure. From Figure 4 it is seen that the majority of the EC-responsible genes are far away from the center region. The average coordinates of A3/(A3+T3) and G3/(G3+C3) was 0.6308 and 0.5729, respectively. Therefore, in support of COA, our PR2 plot analysis further confirms the supremacy of selection pressure in shaping the CUB of EC-responsive genes.

### Gene expression and its relation with various skews:

CAI was used to estimate the expression value of EC-responsible genes. The genes showed CAI value within the range of 0.705 to 0.863, which suggests higher expression. GRAVY analysis revealed that 5% of genes are hydrophobic and 95% of genes are hydrophilic. The aromaticity score can determine stability of the gene. A high aromaticity value signifies a more stable gene structure. 21% of genes show a high aromaticity value. CAI showed a positive correlation of 0.361 and 0.190 (p<0.05) with AT and GC skewness, respectively. Where as, CAI showed a negative correlation with GRAVY and aromaticity score of the EC-responsible genes (GRAVY: r= -0.404, p<0.05; aromaticity: r= -0.357, p<0.05). Amino acid composition analysis revealed that leucine is the highest used amino acid whereas tryptophan is the lowest used amino acid. Serine, alanine, glutamine, and lysine are the frequently used amino acids. Altogether, these findings suggest that the skews played a significant role in modulating the CUB and thereby the gene expression.

### Codon context analysis:

**Figure 5:**
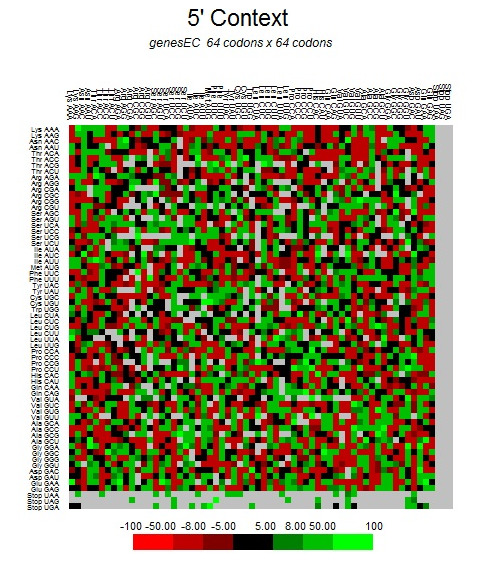
Heat map representing the codon pair context pattern of the esophagus cancer-related genes. Green, black and red-colored dots represents over, absent, and under-represented codon pairs, respectively.

Recently it became evident that not only preference of single codon selection but also the codon pair influences the genes expression and mRNA structure [27,28]. From our analysis, it was observed that 56.73% of codon pairs are over-represented and 26.26% of codon pairs are under-represented. 16.99% of codon pairs are absent in the genes. Heat map for the codon context analysis is shown in Figure 5. The top 10 over-represented codon pairs are GAG-GAG, GAG-CUG, GAG-AAG, CUG-GAG, AAG-AAA, CAG-AAG, GAU-GAC, GAA-GAA, GUG-GAG, and AAA-GAA. Similarly, the top 10 under-represented codon pairs are ACG-GAU, ACG-CGU, ACG-CGG, ACG-CGC, ACG-CAA, ACG-AGG, ACC-CAA, ACA-UCA, ACA-CUA, and ACA-CCA.

## Discussion:

A diverse array of mechanisms regulates protein biogenesis. This phenomenon is more complex in multicellular organisms. A vast range of studies reported the role of transcriptional regulation in disease progression. Here, in the present study, we have done a detailed analysis of the nucleotide composition and their variation resulting in CUB between the genes responsible for the development of esophageal cancer. Next, we did a systematic comparison with the results obtained by other researchers on the same genome or other genomes to identify similarities and differences. This will help to get new molecular insights into esophageal cancer. EC-related genes showed higher use of GC as compared to AT at the synonymous positions as well as in overall gene compositions. Previous reports suggest that the GC-rich genome prefers to use GC-ending codons whereas the AT-rich genome prefers to use AT-ending codons [29]. Therefore, our findings revealed that the nucleotide composition of the EC-related genes followed a similar pattern, corroborated well with the existing CUB reports on human genes [30]. GC composition pattern of a gene greatly influences its codon usage pattern [31]. EC-responsive genes showed an average ENc value of 49.28, which is significantly higher than 35. Extensive research on CUB proposed that ENc>35 should be considered as low CUB [32,33]. In support of the ENc pattern observed in the CNS responsible genes, we observed low CUB of the EC-responsive genes [30]. The overall low CUB observed in multicellular organisms might be related to the replication process as different cell types have different codon preferences [34]. Transcription factor SOX-17 showed the highest CUB whereas, fermitin family homolog 1 showed the least CUB. Interestingly, a significant positive correlation was observed between the GC3 and ENc (r=0.56, p <0.05), suggest genes with higher GC3 have a lower CUB. A similar finding was reported by Malaker et al 2020 in a study conducted on mammalian genes [23]. Furthermore, GC showed strong positive correlations with GC endings codons (Table 2 - see PDF). Therefore, our finding confirmed the relative contribution of GC compositional constraints under the strong mutational pressure in shaping the codon usage pattern of human genes. RSCU analysis revealed the over-representation of GAA, AGA, AGC, GTG, CTG, GAG, and CAG. Similarly the codons GTA, TCG, ATA, TTA, CCG, CGT, ACG, GCG, and CTA as under-represented. 66.66% and 44.44% GC-ending codons were found in over and under-represented codon groups. This confirms the significance of AT nucleotides in modulating the CUB in association with GC compositional constraints. Correspondence analysis identified the contribution of forces behind the observed CUB of EC-responsive genes, where mutation pressure showed dominance over selection pressure. Although a few codons and genes showed scatted distribution i.e. away from the center and major axes, respectively. Cluster analysis supported the COA analysis. In support of the findings reported by Zhang et al [35] here we hypothesized that the observed variation might be due to the prevalence of different EC-responsive genes in different cell types. Mutation pressure causes the proportional use of nucleotides i.e. A=T and G=C. Neutrality and PR2 plot revealed the disproportional use of nucleotides at synonymous codon positions. EC-responsible genes showed higher use of A/G nucleotides, suggest the dominance of selection pressure. Uddin et al observed higher use C at the synonymous codon position for CNS genes [30]. Therefore, the PR2 bias pattern observed in the present study can be used as fingerprints for the EC-responsive genes, which requires further validation and systematic comparison with other diseases. Estimation of the gene expression revealed that all the EC-responsive genes are highly expressive in nature (based on CAI value). A few previous reports confirmed the role of various skews in gene expression [36]. Similarly the EC-responsive genes studied here showed significant relationship with AT-skew, GC-skew, GRAVY, and aromaticity. Furthermore, findings of our codon context analysis revealed that GAN-NNG as the frequent contexts present in EC-responsive genes. Compositional properties of a gene affect the CUB and gene function [37].

## Conclusion:

We show that CAG and GTA are over-represented and under-represented codons, respectively in genes linked with esophageal cancer. Correspondence analysis, neutrality plot, and parity rule 2 plot analysis confirmed the dominance over mutation pressure in modulating the codon usage pattern of genes linked with esophageal cancer.
